# Calcium signals inhibition sensitizes ovarian carcinoma cells to anti-Bcl-x_L_ strategies through Mcl-1 down-regulation

**DOI:** 10.1007/s10495-015-1095-3

**Published:** 2015-01-28

**Authors:** Marie-Laure Bonnefond, Bernard Lambert, Florence Giffard, Edwige Abeilard, Emilie Brotin, Marie-Hélène Louis, Mor Sény Gueye, Pascal Gauduchon, Laurent Poulain, Monique N’Diaye

**Affiliations:** 1 Normandy University, Caen, France; 2 UNICAEN, INSERM U1199 “Biology and Innovative Therapeutics of Locally Aggressive Cancers” Unit, Caen, France; 3 François Baclesse Comprehensive Cancer Center, 3 Avenue du Général Harris, BP5026, 14076 Caen Cedex 05, France; 4 CNRS (placed at the disposition of EA4656 by CNRS), Délégation régionale Ile-de-France Est, 94532 Thiais Cedex, France

**Keywords:** Ovarian cancer, Calmodulin, Mcl-1, Calcium signal, mTOR

## Abstract

Ovarian carcinoma is the leading cause of death from gynecologic cancer in the developed world and is characterized by acquired chemoresistance leading to an overall 5-year survival rate of about 30 %. We previously showed that Bcl-x_L_ and Mcl-1 cooperatively protect platinum-resistant ovarian cancer cells from apoptosis. Despite BH3-mimetics represent promising drugs to target Bcl-x_L_, anti-Mcl-1 strategies are still in pre-clinical studies and required new investigations. Calcium is a universal second messenger and dysregulation of calcium signal is often observed during carcinogenesis. As change in cytosolic free calcium concentration [Ca^2+^]_i_ is known to control the fate of the cell by regulating Bcl-2 family members, we wonder if calcium signal could impact on Mcl-1 expression and if its pharmacological inhibition could be useful to sensitize ovarian carcinoma cells to anti-Bcl-x_L_ strategies. We therefore studied the effect of different calcium signals inhibitors in ovarian carcinoma cell lines SKOV3 and IGROV1-R10 and analysed their effects on proliferation and Mcl-1 expression. We also exposed these cells to these inhibitors in combination with anti-Bcl-x_L_ strategies (siRNA or BH3-mimetic: ABT-737). We found that calcium signaling regulates Mcl-1 through translational events and a calmodulin-mediated pathway. BAPTA-AM and calmodulin inhibitor combination with ABT-737 leads to apoptosis, a process that is reversed by Mcl-1 enforced expression. As Mcl-1 represents a crucial hurdle to the success of chemotherapy, these results could open to new area of investigation using calcium modulators to directly or indirectly target Mcl-1 and thus efficiently sensitize ovarian carcinoma cells to anti-Bcl-x_L_ strategies.

**Electronic supplementary material:**

The online version of this article (doi:10.1007/s10495-015-1095-3) contains supplementary material, which is available to authorized users.

## Introduction

Ovarian cancer causes more than 140 000 deaths worldwide every year, and is the most lethal gynaecological malignancy in developed countries [1]. Standard treatment includes debulking surgery and subsequent platinum-based chemotherapy. Despite good initial response rates, acquired chemoresistance is a critical hurdle in the treatment of cancer and is responsible for relapse and leads to an overall survival rate of about 30 %. One major mode by which cancer cells evade chemotherapy is by an acquired ability to suppress intrinsic pathway of apoptosis [1]. The key regulators of apoptosis are the proteins of the Bcl-2 family which are classified in three groups: the anti-apoptotic proteins (Bcl-2, Bcl-x_L_, Mcl-1…) that sequester the multi-domain pro-apoptotic members group (Bax and Bak) preventing their oligomerization and subsequent apoptosis. The last group consists of the BH3-only members (Bad, Bid, Bim, Noxa, Puma…) those possess only the BH3 domain and act as sensors of cellular stress. They initiate apoptosis by either blocking the activity of anti-apoptotic members or directly activating pro-apoptotic members, which is mediated via interaction of the BH3 domain of one protein with the hydrophobic pocket of another [2].

Overexpression of Bcl-2 family members has been observed in a variety of cancers and contributes to chemoresistance. Actually, Bcl-x_L_ and Mcl-1 are often overexpressed in ovarian carcinoma and are associated with poor prognosis in these cancers [3, 4]. Although the down-regulation of either Bcl-x_L_ or Mcl-1 remains ineffective, we showed that the concomitant inhibition of these proteins with siRNA was sufficient to induce massive cell death, highlighting the pivotal role these anti-apoptotic proteins play in chemoresistant ovarian carcinoma and mesothelioma [5].

Several strategies have been developed to overcome Bcl-x_L_ and BH3-mimetic drugs represent an exciting development in cancer therapeutics. Among these molecules, ABT-737 is the most promising candidate to clinic. It mimics the direct binding of Bad to Bcl-2, Bcl-x_L_ and Bcl-w, with a high-affinity (K_i_ < 1 nM). However, it does not bind to Mcl-1 and Bfl-1 [6] that prevents it to induce cell death in tumor models that overexpress these anti-apoptotic proteins, such as ovarian cancer. Therefore, the use of Mcl-1 targeting agents in combination with BH3-mimetics could constitute an interesting therapeutic strategy in these types of cancers [7].

Many strategies have been developed to counteract Mcl-1 expression or activity such as RNA interference, Noxa overexpression [7] or platinum salts [8]. Mcl-1 is finely regulated and targeting canonical signaling pathways with kinases inhibitors such as PI3K/AKT/mTOR and RAF/MEK/ERK inhibitors gave promising results and strongly sensitized cancer cells to ABT-737 [9, 10]. Concomitant inhibition of these pathways is however required to avoid molecular crosstalks and to induce massive apoptosis [11]. The complexity of pathways controlling Mcl-1 and their interconnectivity encourage research to find other networks that could have a therapeutic interest.

Calcium is a universal second messenger that regulates a number of diverse cellular processes including cell proliferation, apoptosis, motility and secretion. The versatility of the signals is most strikingly exemplified by their role in life-and-death decisions. Consequently, [Ca^2+^]_i_ needs to be used in an appropriate manner to determine cell fate; if calcium signals are compromised, pathologies as carcinogenesis may occur. Actually, modifications of calcium pumps and channels are regularly observed in cancer and have impact on cellular proliferation by activating survival pathway or preventing apoptosis [12]. For example, inhibition of calcium permeable TRPC3 (transient receptor potential canonical type-3) channels, which are elevated in clinical epithelial ovarian cancer samples, reduces the proliferation of SKOV3 ovarian cancer cells [13]. The impact of [Ca^2+^]_i_ on apoptosis regulation could be explained by the tight relation between calcium homeostasis and Bcl-2 family members. Indeed, it has been described that anti-apoptotic members reduced apoptotic signals by allowing continuous calcium leak through endoplasmic reticulum leading to a reduced in amplitude calcium signal which prevented apoptosis trigger. On the contrary, calcium signal can modulate these proteins expression and increase in [Ca^2+^]_i_ was described to induce Bcl-2 expression through CREB (Calcium Response Element Binding protein) activation [14].

Several scattered examples suggest that calcium signaling has an impact on Mcl-1 expression. Indeed, calcium chelation leads to inhibition of EGFR targets (STAT3, AKT, ERK 1/2) that are known to regulate Mcl-1 [15]. Moreover, thapsigargin which increase intracellular calcium concentration via SERCA inhibition induces Mcl-1 expression in melanoma [16]. At last, KN93 a specific calmodulin kinase inhibitor, decreases Mcl-1 expression in prostate cell lines [17]. Here, we tested if calcium signaling pathway inhibition could have an impact on Mcl-1 expression in chemoresistant ovarian carcinoma cells and we evaluated the impact of its combination with anti-Bcl-x_L_ strategies.

## Materials and methods

### Cell culture

SKOV3 was obtained from American Type Culture Collection (Manassas, USA). Resistant IGROV1-R10 cells were obtained by reiterating treatments with increasing concentrations of cisplatin as previously described [10]. Cell lines were grown in RPMI-1640 medium supplemented with 2 mM Glutamax™, 25 mM HEPES, 10 % fetal calf serum, and 33 mM sodium bicarbonate (Fisher Scientific Bioblock, France). Cells were maintained in a 5 % CO_2_ humidified atmosphere at 37 °C.

### Reagents

ABT-737 was purchased by Selleckem (Souffelweyersheim, France) and BAPTA-AM was purchased from Sigma-Aldrich (Saint-Quentin Fallavier, France). W7, KN93, FIPI and z-VAD were provided by Tocris (R&D Systems, Lille, France). These compounds were commonly stored as stock solutions in DMSO at −20 °C. Bortezomib (Velcade^®^) was supplied by Millennium Pharmaceuticals (Cambridge, MA, USA).

### Proliferation analysis

Cell number and viability were estimated by a semi-automated image-based cell analyzer (Cedex XS Analyzer, Roche Applied Science, Meylan, France) using the Trypan blue exclusion method.

### Cell cycle analysis by flow cytometry

Adherent and floating cells were pooled, washed with phosphate-buffered saline (PBS 1X) and fixed with ethanol 70 %. Cells were then centrifuged at 2,000 r.p.m. for 5 min and incubated for 30 min at 37 °C in PBS 1X, to allow the release of low-molecular weight DNA. Cell pellets were stained with propidium iodide using the DNA Prep Coulter Reagent Kit (Beckman-Coulter, France). Samples were analysed using Gallios flow cytometer (Beckman Coulter, France).

### Western-immunoblotting

Cells were rinsed with ice-cold PBS 1X and lysed in RIPA buffer as previously described [8]. After centrifugation, proteins were quantified using the Bradford assay (Bio-Rad, CA). 30 μg of proteins were separated by SDS–PAGE (Biorad, France) and transferred to PVDF-membranes (Millipore, France). After blocking, membranes were incubated overnight at 4 °C with the following primary antibodies: anti-Mcl-1 (Santa Cruz Biotechnology, France), anti-Noxa (Calbiochem, France), anti-Bcl-2 (DAKO, France), anti-actin (Sigma, Saint Louis, USA), anti-Bcl-x_L_, PARP, Bim, caspase-3, p-AKT(thr308), p-AKT(ser473), AKT, p-ERK(Thr202/Tyr204), ERK, p-4E-BP1(Thr70), 4E-BP1, p-p70S6K(Thr389) and p70S6K (Cell Signaling Technology, Ozyme, France). Membranes were then incubated with the appropriate horseradish peroxidase-conjugated secondary antibodies (GE Healthcare, France). Revelation was done using ECL Prime Western Blot detection reagent (GE Healthcare, France).

### RNA extraction and real-time quantitative reverse transcription PCR (qRT-PCR)

Total RNA were isolated from ovarian carcinoma cell lines using Trizol (Invitrogen, Life Technologies, France). RNA quantity and quality were assessed using the NanoDropTM 2000 spectrophotometer (Thermo Scientific, France). The first strand cDNA was synthesized using Omniscript reverse transcriptase kit (Qiagen, France) with random hexamers. cDNA (25 ng) were combined with 10 µmol/l of each forward and reverse primers, 50 µmol/l of the Taq-Man^®^ probe and TaqMan^®^ Fast Universal PCR Master Mix (Applied Biosystems, Foster City, USA) in a 20 µl final reaction volume. Corresponding custom inventoried (ID: Hs00172036_m1 for Mcl-1 and Hs99999905_m1 for GAPDH) TaqMan^®^ Gene Expression Assays were used (Applied Biosystems). All PCR amplification reactions were carried out in triplicate and detection was done on an Applied ABI Prism 7500 Fast PCR system (Applied Biosystems). GAPDH was used as a housekeeping reference gene for normalization.

### siRNA tranfection

PAGE purified siRNAs were synthesized and annealed by the Eurogentec Company (Liège, Belgium). Specific double-stranded 21 nt RNA oligonucleotides forming a 19 bp duplex core with 2 nt 3′ overhangs were used to silence Bcl-x_L_ expression (5′-auuggugagucggaucgca-3′, noted siXL). siGENOME Non-Targeting siRNA Pool#1 (noted siCT) was purchased from Dharmacon. siRNA duplexes were transfected using the INTERFERin™ transfection reagent according to the manufacturer’s instructions (Polyplus-Transfection, France). Briefly, cells were seeded in 25 cm^2^ flasks to reach 30–50 % of confluence at the time of transfection. The transfection reagent and the siRNAs were mixed and complex formation was allowed to proceed for 15 min at room temperature before to be applied to cells. After indicated time, cells were trypsinized and washed with ice-cold PBS 1X before to be analyzed.

### Plasmid construction and transfection

The MCL1 ORF (NM_021960.4, +209 to +1298) was amplified from IGROV-R10 cDNA by PCR using restriction enzyme containing-primers (sense 5′ TTAAGCTTATGTTTGGCCTCAAAAGAAA3′; antisens 5′ GGTCTAGAGGTTGGTTAAAAGTCAACTATTGC 3′). The EIF4E ORF (NM_001968, +1519 to +2222) was amplified from IGROV-R10 cDNA by PCR using restriction enzyme containing-primers (sense 5′ AACAAGCTTCTAAGATGGCGACTGTCGAA 3′; antisens 5′ AACAAGCTTCTAAGATGGCGACTGTCGAA 3′). The amplified ORF were restricted with HindIII and XbaI and cloned between the corresponding restriction sites of the mammalian expression vector pcDNA3.1. The sequences of the construction were checked. Empty vector pcDNA (named “Empty”) and Vector containing MCL1 ORF or EIF4E (named “Mcl-1” and “eIF4E” respectively) were transfected using JetPrime DNA transfecting agent (Polyplus-Transfection, France) as described by manufacturer.

### Real-time cellular cytotoxicity assay (xCELLigence)

Compound-mediated cytotoxicity was monitored using Real-Time Cell Analyzer Multi-Plate (RTCA MP) Instrument, xCELLigence System (ACEA, Ozyme, France). The system monitors cellular events in real time measuring electrical impedance across interdigitated micro-electrodes integrated on the bottom of tissue culture E-plates View. The increase in the number and size of cells attached to the electrode sensors leads to increased impedance, from which derive the cell index (CI) values displayed at the plot, reflecting changes in cell viability. Briefly, 96-well E-Plates were seeded and placed onto the RTCA MP in a tissue culture incubator. Treatments were realized 24 h later and impedance was continuously measured. Standard deviations of well replicates were analyzed with the RTCA Software.

### Statistical analysis

Relative values were compared to the value of the control condition by a one-sample Student’s *t* test.

## Results

### Cytostatic effect of calcium chelator BAPTA-AM

**Fig. 1:**
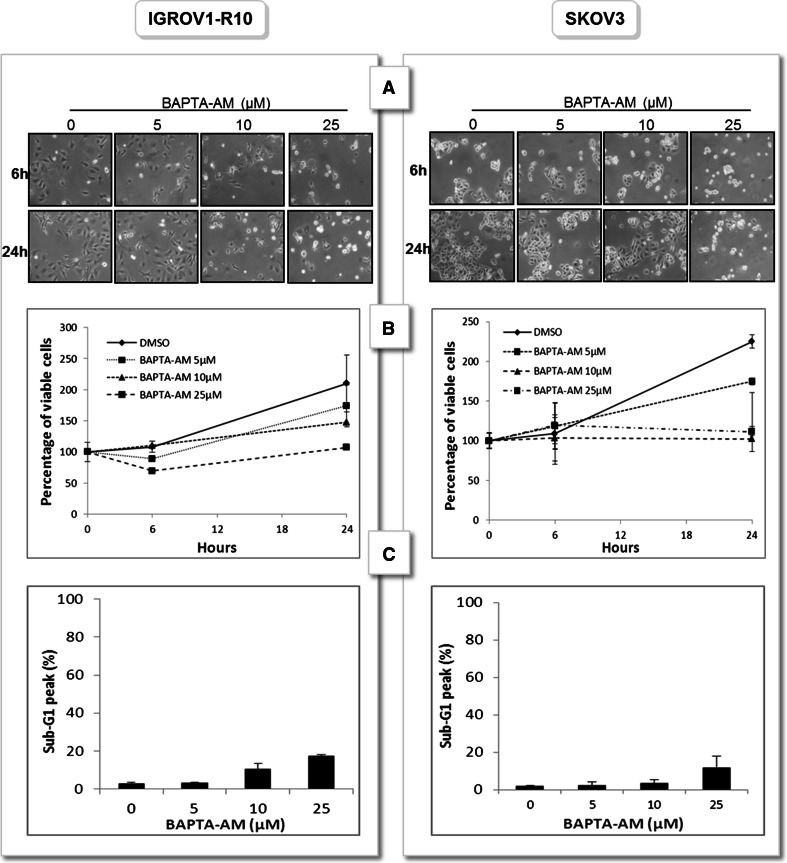
Cytostatic effect of calcium chelator BAPTA-AM. IGROV1-R10 and SKOV3 cells were treated or not (DMSO) with increasing concentrations of BAPTA-AM for 6 and 24 h. Response was appreciated by **a** morphological features **b** cell viability (assessed by the percentage of cell viability with respect to number of viable cells at 0 h) **c** assessment of sub-G1 peak (24 h). Data are representative of three independent experiments

SKOV3 and IGROV1-R10 were treated with increasing concentrations of BAPTA-AM for 6 and 24 h. Results revealed that BAPTA-AM had a dose dependent anti-proliferative effect that appeared from the dose of 10 µM as assessed by morphological features and cell viability for the two lines tested (Fig. 1a, b). The strongest dose tested (25 µM) induced shape modification of cells that became rounded and this effect was observable as soon as 6 h of treatment. Increase in sub-G1 peak was dose-dependent but remains modest even for the highest concentration of BAPTA-AM tested (Fig. 1c).

### BAPTA-AM inhibits Mcl-1 expression

**Fig. 2:**
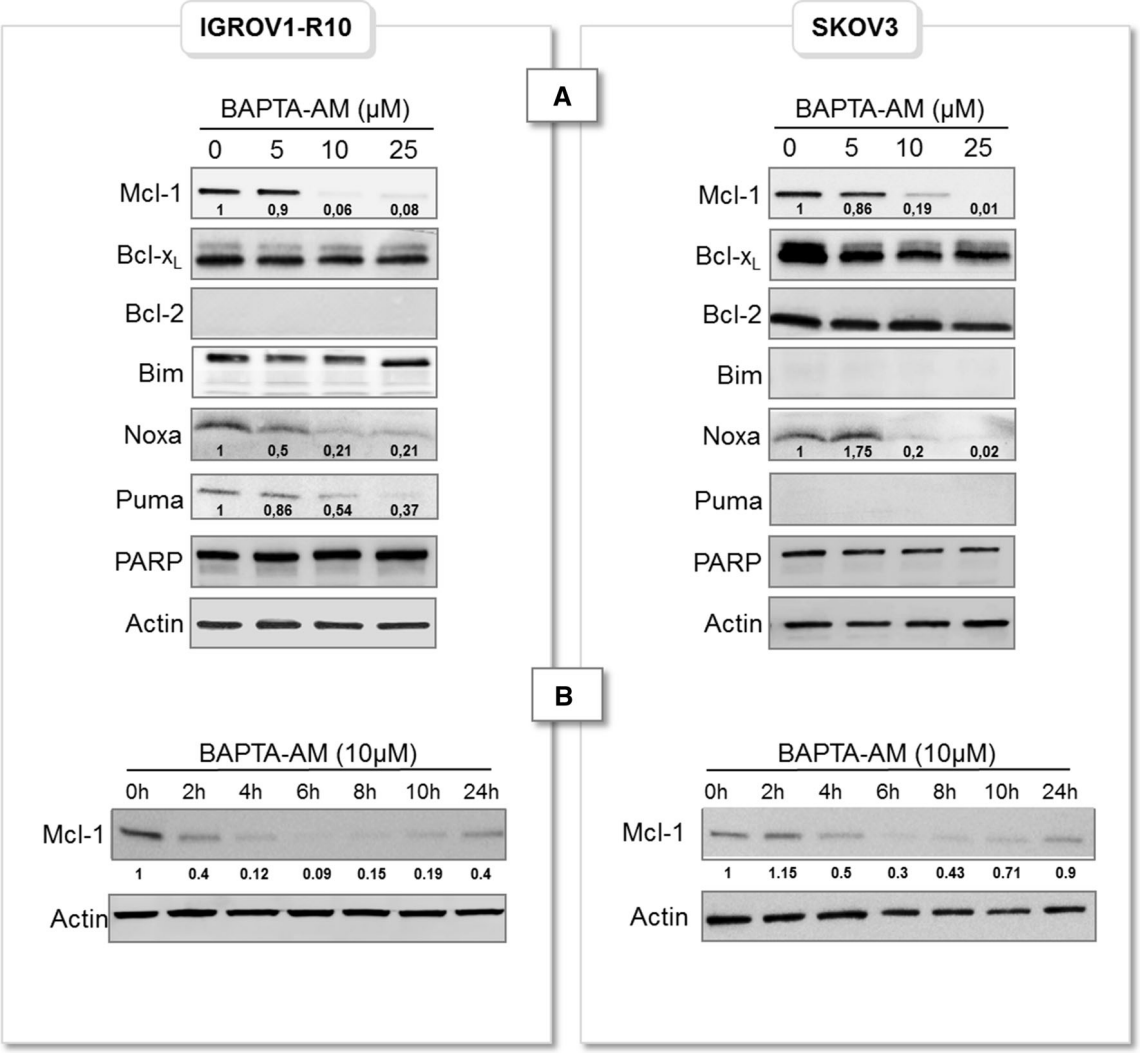
Dose-response and time course of BAPTA-AM-induced Mcl-1 decrease. **a** IGROV1-R10 and SKOV3 cells were treated or not (DMSO) with increasing concentrations of BAPTA-AM for 6 h and expressions of Bcl-2 family members were appreciated by western blot and Mcl-1, Noxa and Puma expressions were quantified by Image J software. **b** IGROV1-R10 and SKOV3 cells were treated with 10 µM BAPTA-AM from 0 to 24 h. Expression of Mcl-1 was assessed by western blot. Data are representative of three independent experiments. Mcl-1 expression was quantified by Image J software. The relative intensity of each *lane* was calculated with respect to the sample at 0 h

IGROV1-R10 and SKOV3 were then treated with increasing doses of BAPTA-AM (0, 5, 10, 25 µM) for 6 h and expression of Bcl-2 family members were analyzed upon this treatment. A deep decrease of Mcl-1 expression appeared from 10 µM in both cell lines (Fig. 2a). Concerning the other members of Bcl-2 family, Bcl-2 was not expressed in IGROV1-R10 and Bim not expressed in SKOV3 cells as previously described [8] however their expression were not modified in the cell line where they are present. As for Puma, this BH3-only was very slightly expressed in IGROV1-R10 cells and its expression also dose-dependently decreased upon BAPTA-AM treatment. This protein was not detected in SKOV3 cells in ours conditions. Noxa was detected in both cell lines and its expression was dose-dependently decreased upon BAPTA-AM treatment.

No PARP cleavage was observed confirming that BAPTA-AM did not induced apoptosis. A time-course experiment with 10 µM BAPTA-AM revealed that Mcl-1 expression dramatically decreased within 6 h but its expression is partially recovered for longer treatment indicating that BAPTA-AM effect is transient (Fig. 2b).

### BAPTA-AM–induced Mcl-1 decrease does not result from transcriptional and post-translationnal events but is associated with mTORC1 pathway inhibition

**Fig. 3:**
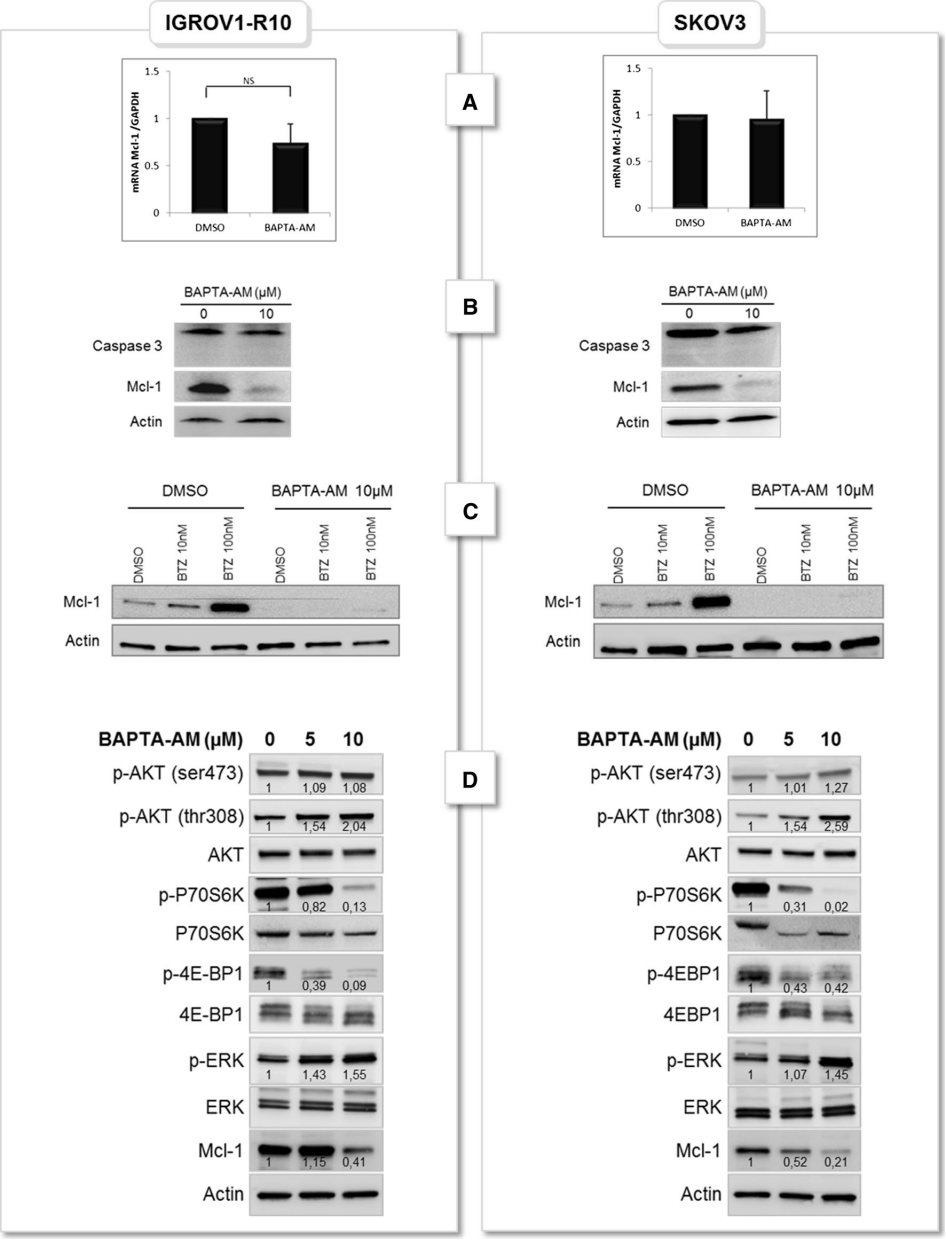
BAPTA-AM–induced Mcl-1 decrease does not result from transcriptional and post-translationnal events but is associated with mTORC1 pathway down-regulation. IGROV1-R10 and SKOV3 cells were treated or not (DMSO) with 10 µM BAPTA-AM for 6 h. **a** Mcl-1 mRNA level was determined by real time quantitative RT-PCR, **b** PARP and Caspase 3 cleavages were assessed by western blot. **c** IGROV1-R10 and SKOV3 cells were pre-treated 1 h with DMSO, 10 or 100 nM bortezomib. Then cells were treated or not (DMSO) with 10 µM BAPTA-AM for 6 h. Expression of Mcl-1 was followed by western blot. Data are representative of three independent experiments. **d** IGROV1-R10 and SKOV3 cells were treated or not (DMSO) with 5 and 10 µM BAPTA-AM for 6 h. AKT/mTOR as well as MAPK pathways were assessed for each condition and proteins expressions were quantified by Image J software. Data are representative of three independent experiments

To decipher the mechanism underlying Mcl-1 down-regulation by calcium inhibition, Mcl-1 mRNA expression in SKOV3 and IGROV1-R10 cells was quantified using RT-qPCR. Treatment of cells with 10 µM BAPTA-AM for 6 h did not significantly altered Mcl-1 at mRNA level (Fig. 3a), suggesting that calcium signal inhibition induced Mcl-1 down-regulation through transcription-independent mechanism. We then tested the involvement of caspase on Mcl-1 stability as Mcl-1 could be degraded by activated caspase 3 [18]. Cells were treated with BAPTA-AM for 6 h and pro- and cleaved- caspase 3 expressions were assessed. No cleavage of caspase 3 was observed allowing us to exclude involvement of caspase in BAPTA-AM–induced Mcl-1 decrease (Fig. 3b).

To analyse if Mcl-1 decrease upon BAPTA-AM treatment involves proteasomal degradation, we incubated ovarian carcinoma cells with bortezomib, a proteasome inhibitor, for 1 h and then treated cells with BAPTA-AM for 6 h. As assessed in Fig. 3c, bortezomib dose-dependently prevented Mcl-1 degradation in SKOV3 and IGROV1-R10 cells. However, this pre-treatment did not prevent the loss of Mcl-1 induced by intracellular calcium chelation, ruling out the involvement of posttranslational events in BAPTA-AM–induced Mcl-1 decrease and strongly suggesting translational events.

To further elucidate mechanisms by which BAPTA-AM might inhibit Mcl-1 translation, we studied the activation of AKT/mTOR pathway. This pathway is the most frequently deregulated pathway in ovarian cancer and it is also known to regulate Mcl-1 translation focusing research to target this network in order to sensitize cancer cells [19]. Results showed that BAPTA-AM had no impact on p-AKT(ser473) but dose-dependently increased p-AKT (thr308) as quantifies by densitometry (Fig. 3D). On the contrary, mTORC1 targets, p-4E-BP1 and p-p70S6K were dose-dependently dephosphorylated in the two cell lines. This result suggests that calcium chelation could inhibit Mcl-1 translation via mTOR pathway inhibition in our models.

Mcl-1 could also be regulated by mitogen activated protein kinase (MAPK) as ERK 1/2 either by transcriptional or by post-translational events both leading to an up-regulation of the anti-apoptotic protein [18]. In order to investigate if Mcl-1 down-regulation was a consequence of p-ERK down-regulation by BAPTA-AM, we evaluated ERK phosphorylation status upon BAPTA-AM treatment. As depicted in western blots, [Ca^2+^]_i_ inhibition led to an increase p-ERK 1/2 expression (1.5×) in both cell lines tested allowing us to rule out involvement of ERK in Mcl-1 down-regulation.

### Calcium chelation combined with anti-Bcl-x_L_ strategies leads to apoptosis in ovarian carcinoma

As Bcl-x_L_ and Mcl-1 cooperates to prevent ovarian carcinoma cells from apoptosis, we next evaluated the efficacy of BAPTA-AM/anti-Bclx_L_ strategies combinations.

**Fig. 4:**
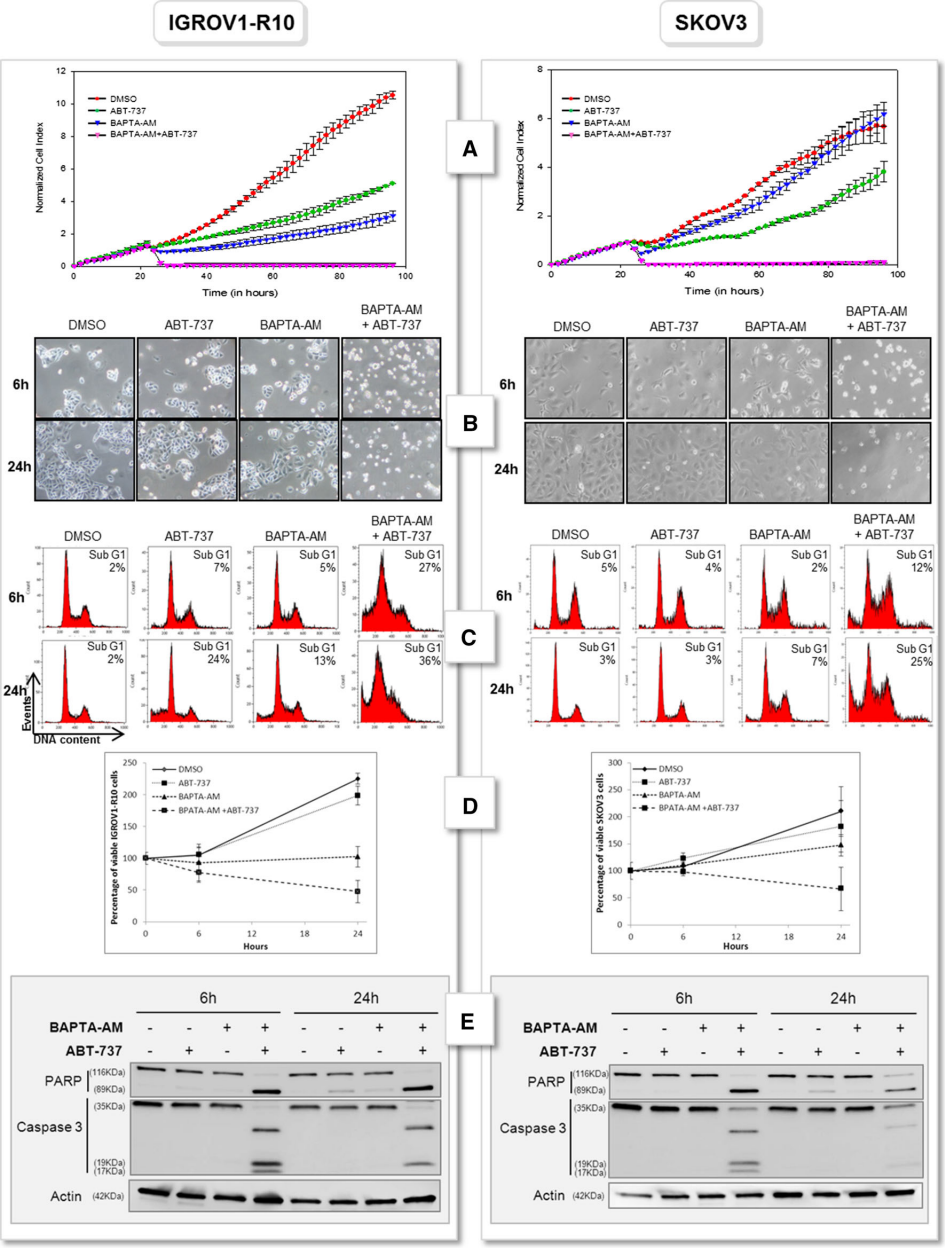
Calcium chelation combined with ABT-737 leads to apoptosis in ovarian carcinoma. **a** Real time analysis of cellular cytotoxicity of ABT-737/BAPTA-AM combination. Histogram was obtained using the xCELLigence System as described in “ Materials and methods” section. Cells were grown for 24 h and then treated or not (DMSO) with 10 µM ABT-737 in presence or not (DMSO) of 10 µM BAPTA-AM. Cell index was recorded every 2 h, with displayed standard error bars. IGROV1-R10 and SKOV3 cells were treated or not (DMSO) with 10 µM ABT-737 in presence or not (DMSO) of 10 µM BAPTA-AM for 6 and 24 h. **b** Morphological features and **c** DNA contents were studied for each condition. **d** Cell viability was assessed by trypan blue exclusion at 24 h. **e** PARP and caspase 3 cleavages were studied by western-blot. Data are representative of three independent experiments

First, we combined BAPTA-AM with the BH3-mimetic ABT-737. We co-treated ovarian carcinoma cells with the two molecules and analysed cell viability by xCELLigence Technology (Fig. 4a). Results showed that treatment with ABT-737 or BAPTA-AM alone slowed down SKOV3 and IGROV1-R10 proliferation compared to DMSO treatment. Conversely, combination of these drugs led to a dramatic drop in cell index (CI). Actually, CI fell to 0 with 6 h of treatment for both ovarian cell lines. This effect is correlated with appearance of floating cells (Fig. 4b), a strong increase of sub-G1 peak (36 % for IGROV1-R10 and 25 % for SKOV3 cells after 24 h of treatment, Fig. 4c) and a dramatic decrease in cell viability (48 % for IGROV1-R10 and 67 % for SKOV3 cells, Fig. 4d). These events were accompanied by caspase 3 and PARP cleavages (Fig. 4e).

To confirm this result, we combined BAPTA-AM with siRNA targeting Bcl-x_L_ (siXL) (Supp data 1A). For this purpose, SKOV3 and IGROV1-R10 cells transfected with siXL for 48 h and then treated with 10 µM BAPTA-AM. The treatments efficacy was followed by xCELLigence Technology. Results showed a dramatic decrease of cell index upon siXL/BAPTA-AM treatment in IGROV1-R10 cells and to a lesser extent in SKOV3 cells. Cell culture was also carried out and cells were transfected 48 h with siRNA and then treated 6 h with BAPTA-AM. Results revealed that whereas a modest apoptosis was obtained with siXL (siXL+DMSO) or BAPTA-AM (siCT+BAPTA-AM) alone, a massive cell death appeared with siXL/BAPTA-AM combination as assessed by morphological features (Supp data 1B). Moreover siXL/BAPTA-AM combination led to a strong increase of sub-G1 peak (57 % for IGROV1-R10 and 30 % for SKOV3 cells Supp data 1C) and a dramatic decrease in cell viability (49 % for IGROV1-R10 and 54 % for SKOV3 cells Supp data 1D) and to PARP and Caspase 3 cleavages (Supp data 1E).

To verify that caspases were involved in BAPTA-AM+ABT-737 combination-induced apoptosis, we pre-treated SKOV3 cell line with a pan caspase inhibitor z-VAD and then exposed cell to the combination of drugs for 6 h. As depicted in Supp. data 2, the strong sub-G1 peak and caspase 3 cleavage induced by this combination were totally abolished upon z-VAD pre-treatment.

### PLD inhibition does not trigger Mcl-1 down-regulation

In order to decipher the molecular pathways that could connect [Ca^2+^]_i_ and Mcl-1, we tested the effect of inhibitors of proteins known to be regulated by [Ca^2+^]_i_ on Mcl-1 expression.

As Ballou and coworkers described in RAT-1 fibroblasts, an increase in [Ca^2+^]_i_ can lead to PLD activation that in turn could activate mTOR and its downstream targets 4E-BP1 and p70S6K [20] It could then be hypothesized that inhibiting PLD could lead to Mcl-1 inhibition in our models. To answer this question, we treated ovarian carcinoma cells with increasing concentrations of 5-fluoro-2-indolyl des-chlorohalopemide (FIPI) a PLD pharmacological inhibitor for 6 and 24 h. The results presented in Supp. data 3A and 3B showed that FIPI did not modify Mcl-1 expression whatever the time and the FIPI concentration considered. Besides, a 6-h treatment with FIPI had no effect on AKT, mTOR, p70S6K and 4E-BP1 phosphorylations (Supp. data 3A). Moreover, FIPI combination with ABT-737 was also performed in IGROV1-R10 and SKOV3 cells lines and followed with xCELLigence technology (Supp. data 3C). Results revealed that FIPI+ABT-737 treatment only slowed down the proliferation of carcinoma cells lines but had not a cytotoxic effect. These results suggest that the calcium/PLD/mTOR pathway does not seem to be involved in Mcl-1 regulation.

### W7, a calmodulin inhibitor, decreases Mcl-1 expression and its combination with ABT-737 is cytotoxic

**Fig. 5:**
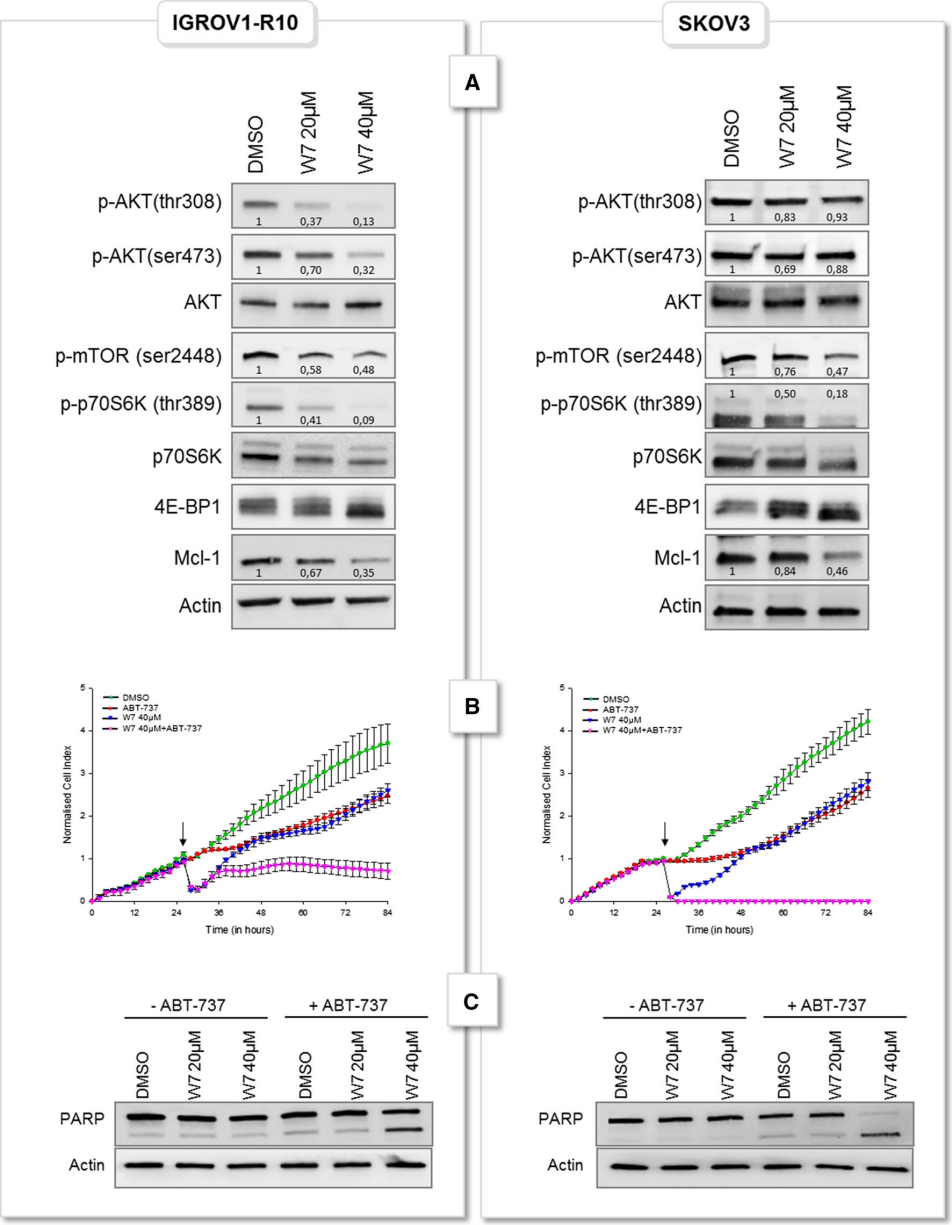
Calmodulin antagonist W7 inhibits Mcl-1 expression and sensitizes ovarian carcinoma cells to ABT-737. **a** IGROV1-R10 and SKOV3 cells were treated or not (DMSO) with increasing concentrations of W7 for 3 h and expressions of Mcl-1 and AKT/mTOR pathway were appreciated by western blot and proteins expressions were quantified by Image J software. Data are representative of three independent experiments. **b** Real time analysis of cellular cytotoxicity of ABT-737/W7 combination. Histogram was obtained using the xCELLigence System as described in “Materials and methods” section. Cells were grown for 24 h and then treated or not (DMSO) with 10 µM ABT-737 in presence or not (DMSO) of 40 µM W7. Cell index was recorded every 2 h, with displayed standard *error bars*. **c** IGROV1-R10 and SKOV3 cells were treated or not (DMSO) with 10 µM ABT-737 in presence or not (DMSO) of increasing concentrations of W7 for 24 h. PARP cleavage was studied by western-blot. Data are representative of three independent experiments

To further examine whether calcium mediates its effect on Mcl-1 through the calcium sensor calmodulin, we used a selective calmodulin antagonist, W7. As shown in Fig. 5a, 3-h treatment with W7 dose dependently decreased Mcl-1 expression in both cell lines. This effect was accompanied with a decrease of mTOR, p70S6K and 4E-BP1 phosphorylation. W7 also caused significant inhibition of AKT (thr308) and (Ser473) phosphorylations in IGROV1-R10 cells whereas it had not effect on AKT phosphorylation in SKOV3 cells.

Then, we combined W7 with the BH3-mimetic ABT-737 and analysed cell viability by xCELLigence Technology (Fig. 5b). Results showed that treatment with 10 µM ABT-737 or 40 µM W7 alone slowed down SKOV3 and IGROV1-R10 proliferation compared to DMSO treatment. Conversely, combination of these drugs led to a dramatic drop in cell index. To confirm this result, we treated cells this W7+ABT-737 combination and observed a strong apoptosis assessed by a decrease of cell viability, increase of sub-G1 peak (data not shown) and appearance of PARP cleavage (Fig. 5c).

### Calcium-calmodulin dependent kinase II (CamKII) is not involved in Mcl-1 down-regulation

Ca(2+)/CaM binds and activates a plethora of enzymes, including Calcium/calmodulin-dependent kinase II (CamKII), calmodulin kinase kinases (CamKK) or AKT [21]. CamKII is ubiquitously expressed and mediated diverse physiological response to increases in [Ca^2+^]_i_ by virtue of its activation by Ca2 +/calmodulin and auto-phosphorylation. CamKII is actually known to activate AKT/mTOR pathway and was demonstrated to down-regulate Mcl-1 in prostate cancer cells [17]. To test the potential involvement of CamKII in Mcl-1 down-regulation in our models, we treated ovarian cell lines with a well-known CamKII inhibitor, KN93. For this purpose, ovarian carcinoma cells were treated with increasing concentration of KN93 for 6 h. Mcl-1 and AKT/mTOR activation were then assessed by western blot and results revealed that KN93 has a modest effect on Mcl-1 (0.93× and 0.82× for 10 µM KN93 in IGROV1-R10 and SKOV3 respectively) and has a modest effect on AKT phosphorylation leading to the conclusion that a calcium/calmodulin/CAMKII pathway does not seem to be involved in Mcl-1 regulation in the conditions tested.

### Enforced expression of Mcl-1 and eIF4E rescue ovarian carcinoma cells from apoptosis triggered by calcium inhibitors + ABT-737 combinations

**Fig. 6:**
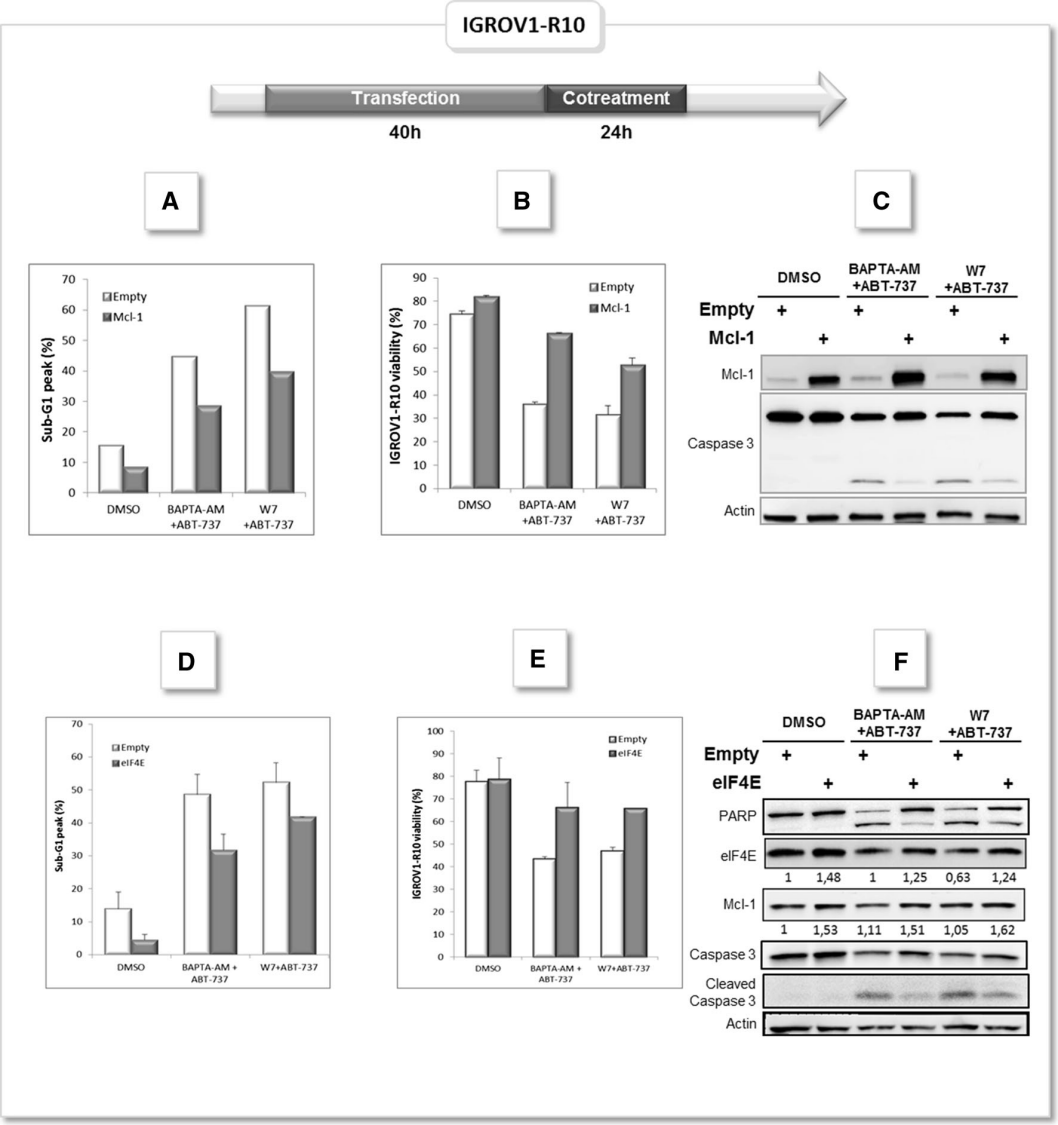
Mcl-1 enforced expression rescue ovarian carcinoma cells from apoptosis triggered by BAPTA-AM/ABT-737 or W7/ABT-737 combinations. IGROV1-R10 cells were transfected with empty plasmid pcDNA (Empty) or pcDNA containing Mcl-1 ORF (Mcl-1) for 40 h as described in “Materials and methods” section. Then cell were treated with DMSO or cotreated either with 10 µM BAPTA-AM and 10 µM ABT-737 or 40 µM W7 and 10 µM ABT-737 for 24 h. **a** DNA contents were studied for each condition. **b** Cell viability was assessed by trypan blue exclusion. **c** Mcl-1 expression and Caspase 3 cleavage were studied by western-blot. IGROV1-R10 cells were transfected with empty plasmid pcDNA (Empty) or pcDNA containing EIF4E ORF (eIF4E) for 40 h as described in “Materials and methods” section. Then cell were treated with DMSO or cotreated either with 10 µM BAPTA-AM and 10 µM ABT-737 or 40 µM W7 and 10 µM ABT-737 for 24 h. **d** DNA contents were studied for each condition. **e** Cell viability was assessed by trypan blue exclusion. **f** Mcl-1, eIF4E expression and PARP and Caspase 3 cleavage were studied by western-blot

In order to assess that down-regulation of Mcl-1 plays a central role in calcium inhibitors + ABT-737-induced apoptosis, we combined BAPTA-AM and W7 with ABT-737 in IGROV1-R10 cells overexpressing or not Mcl-1. Cells were transfected 40 h with empty pcDNA (Empty) or pcDNA containing Mcl-1 cDNA sequence (Mcl-1) as described in “Materials and methods” section and then treated with each combination for 24 h. Results presented in Fig. 6 suggest that enforced expression of Mcl-1 partially rescues ovarian carcinoma cell from calcium inhibitors + ABT-737-induced cytotoxicity as assessed by the decrease of the percentage of sub-G1 peak (45 vs 28 % for BAPTA-AM+ABT-737 condition and 61 vs 40 % W7+ABT-737 condition) (Fig. 6a), the increase of IGROV1-R10 cell viability (36 vs 66.5 % for BAPTA-AM+ABT-737 condition and 32 vs 53 % W7+ABT-737 condition) (Fig. 6b) and the reduction of Caspase 3 cleavage (Fig. 6c).

To confirm that BAPTA-AM and calmodulin regulate Mcl-1 through mTORC1 pathway, we overexpressed 4E-BP1 target eIF4E (eukaryotic translation Initiation Factor 4E). Actually, inactivation of the translational repressor 4E-BP1 by phosphorylation facilitates the detachment and subsequent phosphorylation of eIF4E. Phosphorylated eIF4E in turn forms a complex with eIF4A and eIF4G, which enables the translation of cap-dependent mRNA for proteins like Mcl-1 [22]. Cells were transfected 40 h with empty pcDNA (Empty) or pcDNA containing eIF4E cDNA sequence (eIF4E) and then treated with BAPTA-AM or W7+ABT-737 for 24 h. As presented in Fig. 6, enforced expression of eIF4E partially rescues ovarian carcinoma cell from calcium inhibitors + ABT-737-induced cytotoxicity as assessed by the decrease of the percentage of sub-G1 peak (48.6 vs 31.7 % for BAPTA-AM+ABT-737 condition and 52.3 vs 41.8 % W7+ABT-737 condition) (Fig. 6d), the increase of IGROV1-R10 cell viability (43.4 vs 66.3 % for BAPTA-AM+ABT-737 condition and 47.1 vs 65.7 % W7+ABT-737 condition) (Fig. 6e), an increase in Mcl-1 expression (1.5× in average) and the reduction of Caspase 3 and PARP cleavages (Fig. 6f).

## Discussion

In this study, we showed that BAPTA-AM had an anti-proliferative effect on its own from the dose of 10 µM. For the strongest concentration (25 µM) this effect was accompanied with morphological changes of cells that become rounded within 6 h of treatment. This loss of spindle-shape morphology with BAPTA-AM was also seen in breast cancer cells where this chelator promoted N-cadherin inhibition thereby preventing the epithelial-mesenchymal transition induced by EGF [15].

BAPTA-AM treatment induced a strong inhibition of Mcl-1 expression. However response duration is short as Mcl-1 expression move back up after 24 h treatment. This could be due to BAPTA-AM degradation or to the extremely rapid turnover of Mcl-1. Actually Mcl-1 half-life is about 2 h and we previously demonstrated that in our model, its half-life was about 40 min [8] that could overcome BAPTA-AM action. Mcl-1 down-regulation was accompanied by a reduction in Noxa expression. This result was also observed when Mcl-1 is inhibited by AZD8055 [23] or silenced by siRNA mechanism in ovarian carcinoma cells [8] and melanoma cell lines [24] however further studies are required to understand this mechanism.

Mcl-1's own Achilles’ Heel may lie in its tight regulation and because calcium can affect multiple pathways, we sought how it impacts on Mcl-1 expression. As induction of [Ca^2+^]_i_ by thapsigargin has been described to increase Mcl-1 transcription in melanoma [16], we evaluated if calcium modulation could target Mcl-1 transcription in our models; however results suggest that this mechanism does not seem to be involved in ovarian carcinoma cells.

Mcl-1 down-regulation can also be triggered by two post-translational pathways. The first one was attributed to the proteasome after ubiquitination by Mcl-1 Ubiquitin Ligase E3 (MULE) or beta transducin-containing protein (beta-TrCP). Secondly, Mcl-1 is known to be additionally subject to non-proteasomal degradation and cleaved by caspase 3 at two sites within the N-terminus [18]. However, in our condition neither proteasomal nor caspase 3 degradation were involved in Mcl-1 inhibition and this strongly supports that BAPTA-AM down-regulates Mcl-1 through translational events.

We therefore analyzed if this effect could imply the canonical PI3K/AKT/mTOR pathway that is the most frequently deregulated pathway in high grade serous ovarian cancer. This pathway is all the more interesting that it is known to regulate Mcl-1 translation and is targeted in this way to sensitize several types of cancer justifying the development of pharmacological PI3K/AKT/mTOR inhibitors [10, 19, 25]. Moreover, potential connections between calcium signaling and PI3K/AKT/mTOR pathway have been reported in a number of cell types included ovarian cancer [15, 26, 27]. Results showed that inhibition of Mcl-1 by BAPTA-AM was accompanied by a dramatic p-4E-BP1 and p-p70S6K down-regulation. These proteins are considered as the two most prominent and well-characterized translational regulators activated by mTORC1 and a growing body of evidence suggests that deregulation in protein synthesis has impact on cancer cell survival. In this way, loss of 4E-BP1 phosphorylation has previously been shown to result in a decrease of Mcl-1 protein expression in various cancer types in vitro and in vivo leading to therapeutic benefit [19, 22] and overexpression of eIF4E led to Mcl-1 expression in our models. Our findings suggest that calcium inhibition act on the PI3K/AKT pathway as an mTORC1 inhibitor. This is all the more highlighted because this compound, by inhibiting p70S6K, reactivate PI3K which leads to an increase of p-AKT(thr308). However, our study suggests that calcium inhibition does not act as a classical allosteric mTORC1 inhibitors as rapamycin. Indeed, these latter compounds have been described to poorly inhibit 4E-BP1 phosphorylation and not to target mTORC2 thus leading to an up-regulation of p-AKT(ser473) by a compensatory feedback loop [28]. Yet, p-4E-BP1 expression was strongly inhibited upon BAPTA-AM treatment especially in IGROV1-R10 cells but no increase of p-AKT(ser473) was observed in both ovarian cell lines. This latter result was in agreement with those obtained in human breast cancer cells by Davis FM and coworkers [15]. One could suggest that calcium inhibition act as ATP-competitive inhibitors of mTOR that block the phosphorylation of all downstream targets of mTORC1 and mTORC2 [29]. However, these compounds are known to strongly inhibit p-AKT(ser473) phosphorylation that it is not observed in our models [30, 31].

Concerning RAS/MEK/ERK network, it is known to interfere with PI3K/AKT pathway and regulate mTORC1 activity [32]. It has also been demonstrated that MAPK inhibition led to Mcl-1 down-regulation that sensitized acute myeloid leukemia to ABT-737 action [9]. However, in our conditions, p-ERK was not decreased upon BAPTA-AM treatment but is increased. This effect could be triggered by a negative feedback loop due to mTORC1 inhibition that hyperactivate RTK/IRS/PI3K pathway as reported by Carracedo et al., [30]. This is suggested in our study by the increase of AKT phosphorylation in Thr308. BAPTA-AM induced ERK phosphorylation could also be triggered by calcium dependent regulation of mitogen-activated protein kinase phosphatases, as proposed by Davis FM and coworkers [15]. ERK was described to either to favor cell proliferation or to trigger cell death depending on cell types and stimulus [33, 34]. However, upon BAPTA-AM+ABT-737-induced apoptosis, ERK phosphorylation returned back to the basal level (data not shown). This result shows that up-regulation of ERK phosphorylation is not required when apoptosis occur and that p-ERK does not seem to have a pro-apoptotic action in our models. This is in agreement with the conclusions we already obtained with BEZ-235 [10] and miR-491-5p [35]. Actually, in these studies p-ERK was shown to prevent apoptotic processes due to its capacity to phosphorylate the pro-apoptotic BH3-only Bim leading to its proteasomal degradation.

One hypothesis that could explain the differential regulation of mTOR and AKT is that mTOR regulation could partially be independent of AKT’s one. Conus et al. have shown that calcium regulates differently AKT and p70S6K in Balb/c-3T3 fibroblasts. Actually, they demonstrated that these two kinases are likely to lie on separated pathways with the activation of p70S6K requiring a separate calcium-dependent process [36]. These results were also observed in rat-1 fibroblasts where mTOR and its downstream targets activation were achieved either by PDGF via a classical calcium insensitive PI3K/AKT pathway or by a calcium sensitive phospholipase D/phosphatidic acid pathway [20]. We thus tested if inhibiting PLD could lead to Mcl-1 inhibition. Actually no Mcl-1 expression modification was detected with FIPI in the conditions tested. These treatments modified neither AKT activation nor mTOR, p70S6K and 4E-BP1 phosphorylation (Supp data 3) suggesting that calcium/PLD/mTOR pathway does not seem to be involved in Mcl-1 regulation.

We also tested the potential involvement of Protein Kinase C (PKC) in Mcl-1 down regulation. PKC has been discovered as a calcium and phospholipid dependent serine/threonine-specific protein kinase. The classical PKC isoforms (cPKCs) require calcium for optimal activity. These proteins are involved in several cellular processes, such as cell proliferation, differentiation, and survival, and they are also important for the establishment and progression of malignant disorders such as cancer [37]. At last, Bryostatin (a macrocyclic lactone) or activation of sphingosine-1-phosphate receptor were described to induce Mcl-1 expression through PKC activation [37, 38]. To assess the possible involvement of PKC in calcium-mediated Mcl-1 regulation, we treated ovarian carcinoma cells with a specific PKC inhibitor, GF109203X. A dose response and a time course treatments were performed in both cell lines but no modulation of Mcl-1 expression was observed whatever the dose and the time considered suggesting that calcium-regulated Mcl-1 expression does not require PKC activation (data not shown).

We next tested whether calmodulin antagonists as W7 could down-regulate Mcl-1. Calmodulin is one of the major calcium sensor in the cell and plays central role in cell motility, proliferation and apoptosis [21]. Calmodulin is described to interact directly with numerous proteins as mTOR [39] or AKT [40]. The results obtained showed that W7 decreases Mcl-1 expression and mTOR targets activation in both cell lines. mTOR regulation by calmodulin was often described and molecular mechanisms were elegantly decipher by Gulati [39]. In this study, authors demonstrated that amino acids triggered a rise of [Ca^2+^]_i_ from extracellular stores that bound calmodulin. Thereafter, this complex bound a class III PI3K (human vacuolar protein sorting 34, called hVps34) through its conserved calmodulin-binding motif which triggered mTOR complex activation. It is noteworthy that, like in our study, BAPTA-AM was able to inhibit p70S6K and 4E-BP1 phosphorylations but had no effect on AKT (ser473). It could be hypothesized that similar events occur in ovarian carcinoma cells. As enforced Mcl-1 expression protects from W7 + ABT-737 or BAPTA-AM+ABT-737 apoptosis, this anti-apoptotic protein could be considered as one of the calcium and calmodulin target.

The comparison of W7 effect in the 2 ovarian cell lines tested revealed that W7 differently regulate AKT phosphorylation. Actually, whereas W7 does not modify AKT activation in SKOV3 cells, it strongly decreased phospho-AKT expression in IGROV1-R10 cells. Similar results were also obtained by Coticchia et al., in breast carcinoma cells [41]. In this study, authors found that EGF-induced AKT activation was dependent on calmodulin in the majority of human breast cancer cell lines. However, in some cases this effect did not occur. In their study, this discrepancy partially depends on the basal level of activated AKT because enforced expression of AKT reduced the effect of W7 expression. This does not seem to be the case in our study because basal level of AKT (Thr308) and (Ser473) are higher in IGROV1-R10 cell lines than in SKOV3 (cf [10] ). They also found that forced overexpression of EGFR and ErbB2 partially restores calmodulin-dependent AKT signaling suggesting that EGFR status could explained this discrepancy. However, both ovarian carcinoma cell lines express similar EGFR basal level (data not shown). These hypotheses could not then explain the difference of sensitivity in the cells lines tested and further researches are required to decipher the molecular events involved. However it is important to note that whatever was the sensitivity of AKT to W7 in breast cancer cell lines tested, BAPTA-AM treatment was never able to inhibit AKT activation [41].

A plausible hypothesis that explains why AKT is sensitive to calmodulin and not BAPTA-AM is that its activation could be mediate via the action of a calcium-independent calmodulin (apo-calmodulin) in IGROV1-R10. Actually, apo-calmodulin is a protein that differs from calcium-bound calmodulin in its tertiary structure, and like calcium-bound form, is known to be involved in functions vital to cellular life. These results are not however in agreement with Deb TB and coworkers study where BAPTA-AM and W7 both inhibit EGF-induced AKT (ser473) phosphorylation, suggesting that in MYC83cells, unlike IGROV1-R10 cells, AKT activation is mediated via a calcium-dependent calmodulin. This discrepancy highlight that calcium-mediated AKT regulation is specific to each cell type tested.

Ca^2+^/calmodulin complex is known to activate many cellular effectors including Calcium/calmodulin-dependent kinase II (CamKII), calmodulin kinase kinases (CamKK) or AKT [21]. To find molecular intermediate between Ca^2+^/calmodulin complex and Mcl-1, we first evaluated if CamKII could be involved in Mcl-1 expression. Actually, this kinase was involved in Mcl-1 regulation in a model of prostate carcinoma cells [17]. Results presented in Supp data 4 revealed that KN93 has a modest effect on Mcl-1 and has a modest effect on AKT phosphorylation leading to the conclusion that a calcium/calmodulin/CAMKII pathway does not seem to be involved in Mcl-1 regulation in the conditions tested. This is not in agreement with Ma S. study where CamKIINβ, an endogenous inhibitor of CamKII was found to down-regulate AKT (Ser 473) expression in HO-8910PM ovarian cancer cells. A hypothesis that could explain this discrepancy is that we treated our cells only for 6 h and it could not be ruled out that KN93 decreases AKT phosphorylation for longer time of treatment (24, 48 and 72 h) as presented by Ma S. and coworkers with CamKIINβ. Moreover CamKIINβ is possibly more specific to CamKII than KN93 is, explaining AKT(ser473) stronger inhibition.

Finally, CAMKK inhibition with STO-609 did not down-regulate Mcl-1 expression suggesting that these kinases were not implicated in calcium/calmodulin-mediated Mcl-1 up-regulation (data not shown).

We previously demonstrated that ovarian carcinoma cells are addicted to Bcl-x_L_ and Mcl-1 anti-apoptotic members and that these two proteins cooperate to compromise chemosensitivity. Actually silencing these proteins with siRNA is sufficient to eradicate ovarian carcinoma cells without the requirement of chemotherapy [5, 8]. Targeting Mcl-1 is all the more crucial that ABT-737 (a powerful Bcl-x_L_ inhibitor which is a potential candidate for clinic use) is not only unable to target this protein but also increases Mcl-1 expression inducing by this way its own chemoresistance. Actually, calcium has a strong impact on cellular fate and calcium signaling is often deregulated during carcinogenesis. Moreover, calcium pathway has already been described to regulate Mcl-1 in other types of cancer [16, 17] and this study suggests that Mcl-1 is also regulated by calcium signaling in ovarian carcinoma cells. To comfort these findings overexpression of 4E-BP1 target: eIF4E (eukaryotic translation Initiator Factor 4E) increased Mcl-1 expression and rescued ovarian carcinoma cells from calcium signaling inhibitors + ABT-737-induced apoptosis suggesting that Mcl-1 is crucial for ovarian carcinoma cell survival and that calcium signals act partially through mTORC1 pathway.

The universality of calcium signaling leads to think that targeting calcium would have major effects on all cell types and have not its place for molecular targeted therapy. Actually, a misconception is that calcium is often seen as a simple switch to trigger cellular responses. However, it appears that according to the cell line tested, modulating calcium does not have the same consequence on AKT/mTOR and ERK activation which supports that calcium signaling is specific to cancer cells type. What could explain these differences is that calcium signaling ensues from channels and pumps that specifically regulate cellular processes controlled by calcium and many of these calcium toolkits exhibit specific tissue distribution and their alteration are considered as cancer signatures [42, 43]. Actually, an elegant work from Dubois et al. [44] demonstrated that prostate cancer can undergo an oncogenic switch due to an increase in ORAI3 expression. This alteration modified the nature of the calcium channel from a store-operated calcium channel (constituted of ORAI1 subunits) to an arachidonic acid-regulated one (ARC channel—constituted of ORAI1/3 subunits) leading to increased proliferation and apoptotic resistance promotion [44]. In a similar way, the nature of TRP calcium channels expression is correlated to clinical parameters in breast cancer. Indeed, the expression of TRPM8 is significantly associated with low histological grade tumors and could be considered as a good prognostic marker; by contrast, TRPV6 overexpression is correlated with high-grade tumors and is regarded as a marker of poor prognosis [42, 45]. Concerning ovarian carcinoma cells, TRPV6, TRPC3 and T-type calcium channel are found to be overexpressed leading to think that these proteins could be considered as therapeutic targets [13, 46, 47]. It is thus possible that according to the calcium toolkits expressed in cancer cells types, calcium signals could differs in amplitude and frequency leading to activation of specific calcium effectors and specific cellular responses.

One interesting thing described by Dziegielewska and coworkers is that calcium channels inhibitors could be therapeutically beneficial when combined with standard cytotoxic chemo- or radiotherapy. Thus already commercialized calcium inhibitors could be diverted to be used in chemotherapy. In this regard, clinical trial combining Mibefradil Dihydrochloride (a known antihypertensive drug) and Temozolomide has been approved for treating patients with recurrent glioma (NCT01480050) [48].

Taken together, we found that calcium signaling regulates Mcl-1 through translational events and a calmodulin-mediated pathway. BAPTA-AM and calmodulin inhibitor combination with ABT-737 leads to apoptosis, a process that is reversed by Mcl-1 enforced expression. As Mcl-1 represents a crucial hurdle to the success of chemotherapy, these results could open to new area of investigation using calcium modulators to directly or indirectly target Mcl-1 and thus efficiently sensitize ovarian carcinoma cells to anti-Bcl-x_L_ strategies.

## Electronic supplementary material

Below is the link to the electronic supplementary material.

Supplementary material 1 (TIFF 800 kb)

Supplementary material 2 (TIFF 226 kb)

Supplementary material 3 (TIFF 329 kb)

Supplementary material 4 (TIFF 198 kb)

Supplementary material 5 (DOCX 13 kb)

## Abbreviations

4E-BP1: Eukaryotic translation initiation factor 4E (eIF4E)-binding protein
BAPTA-AM: 1,2-Bis-(*o*-Aminophenoxy)-ethane-*N*,*N*,*N*’,*N*’-tetraacetic acid, tetraacetoxymethyl ester
[Ca^2+^]_i_: Intracytosolic calcium concentration
CaMKII: Calcium/calmodulin-dependent kinase II
CREB: C-AMP response element-binding protein
EGFR: Epidermal growth factor receptor
eIF4E: Eukaryotic translation initiator factor 4E
ERK 1/2: Extracellular signal-regulated kinase
HA14-1: Ethyl [2-amino-6-bromo-4-(1-cyano-2-ethoxy-2-oxoethyl)]-4H-chromene-3-carboxylate
MEK: Mitogen-activated protein kinase
mTORC1/2: Mammalian target of rapamycin complex 1/2
p70S6K: p70 S6 Kinase
PARP: Poly(ADP-ribosyl) polymerase
PI3 K: Phosphatidylinositol 3-kinase
PKC: Protein kinase C
PLD: Phospholipase D
SERCA: Sarcoplasmic endoplasmic calcium ATPase
TRP: Transient receptor potential
z-VAD: *N*-benzyloxycarbonyl-VAD-fluoromethyl ketone
