# Supplementary figures and images for: Calcium signals inhibition sensitizes ovarian carcinoma cells to anti-Bcl-xL strategies through Mcl-1 down-regulation
Source: Apoptosis. 2015 Jan 28;20(4):535–50. doi: 10.1007/s10495-015-1095-3 (PMC4348506; doi:10.1007/s10495-015-1095-3)

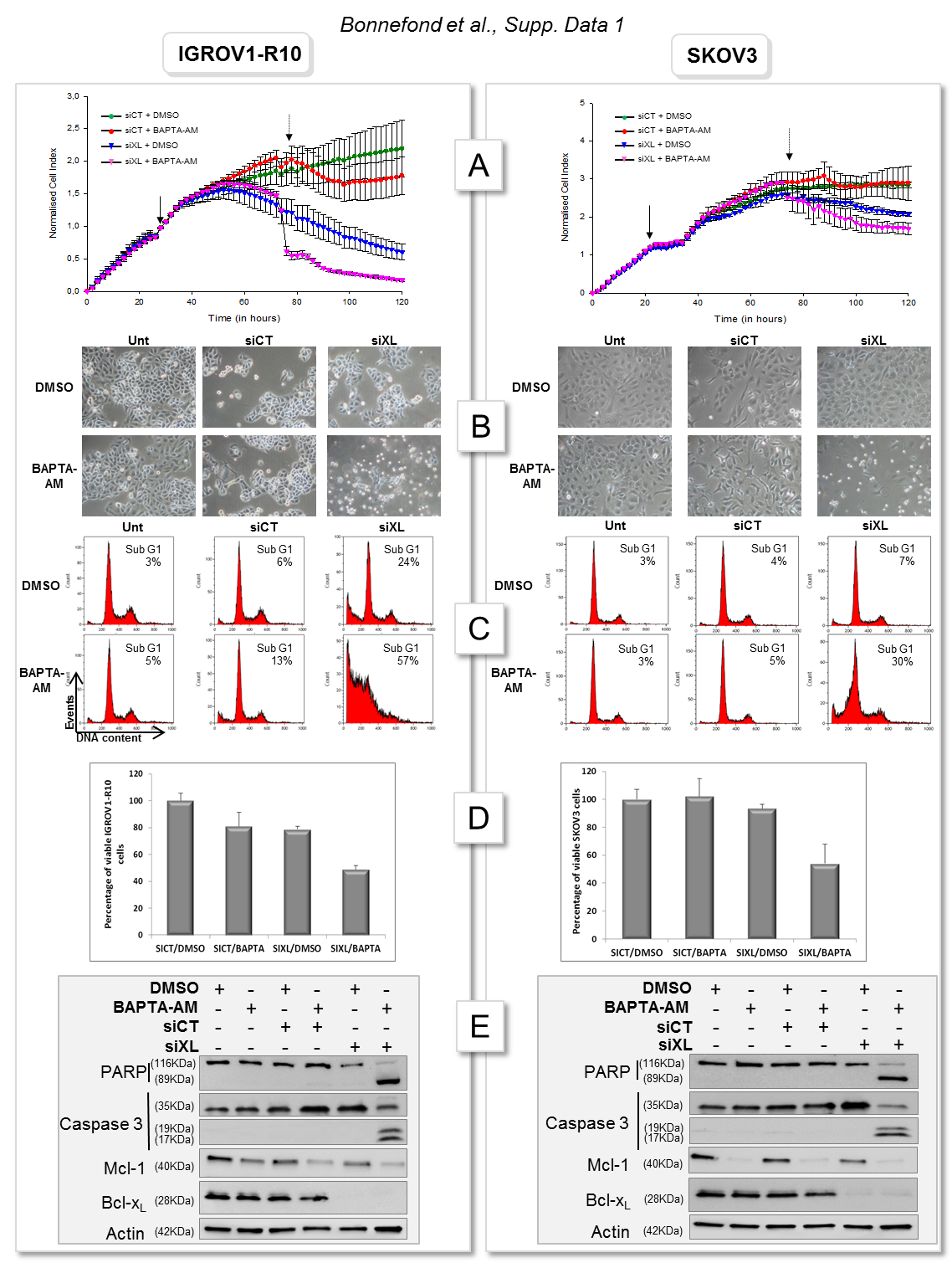

Supplement: Supplementary file 1 — Supplementary material 1 (TIFF 800 kb) [file 10495_2015_1095_MOESM1_ESM.tif]

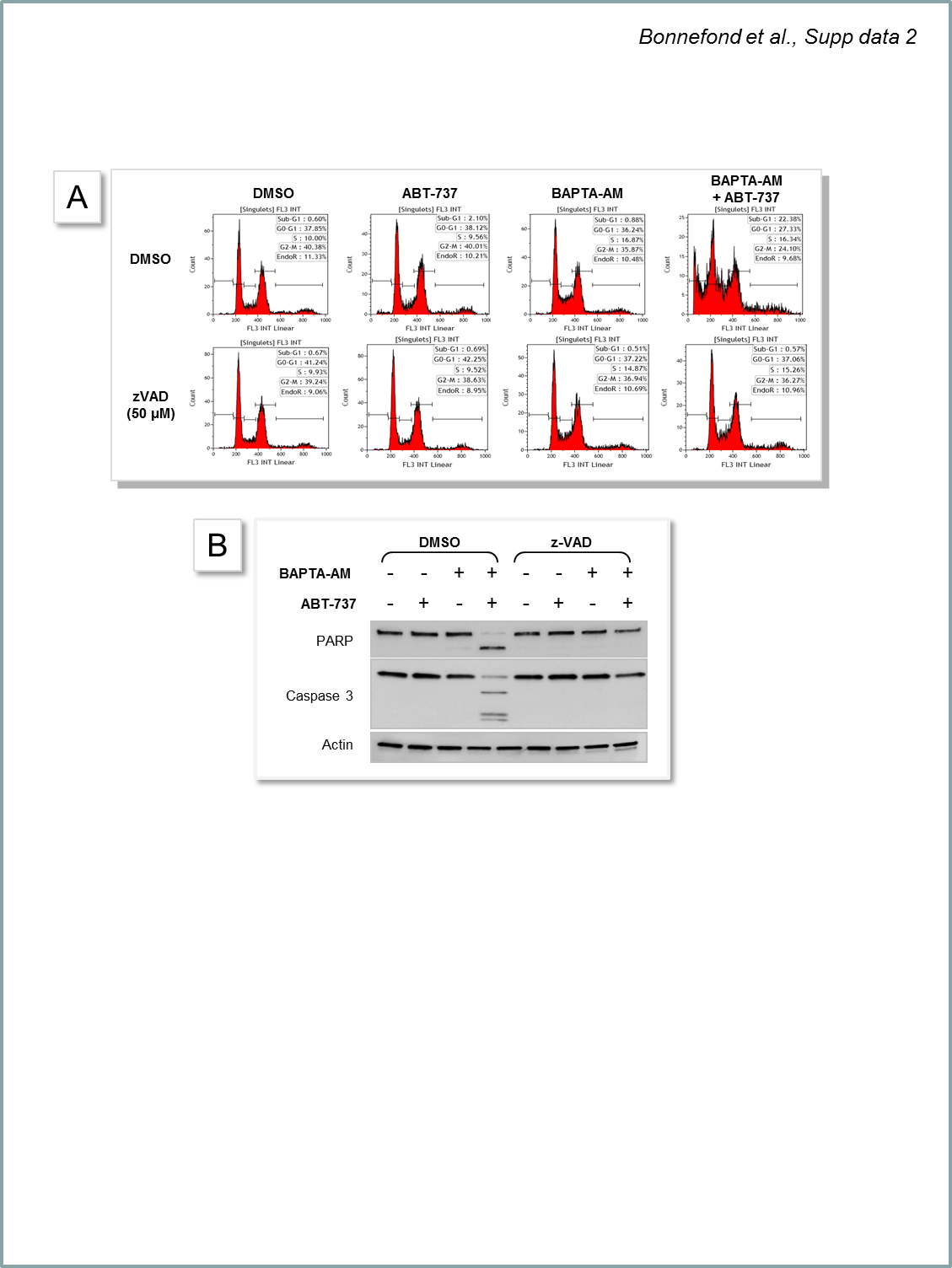

Supplement: Supplementary file 2 — Supplementary material 2 (TIFF 226 kb) [file 10495_2015_1095_MOESM2_ESM.tif]

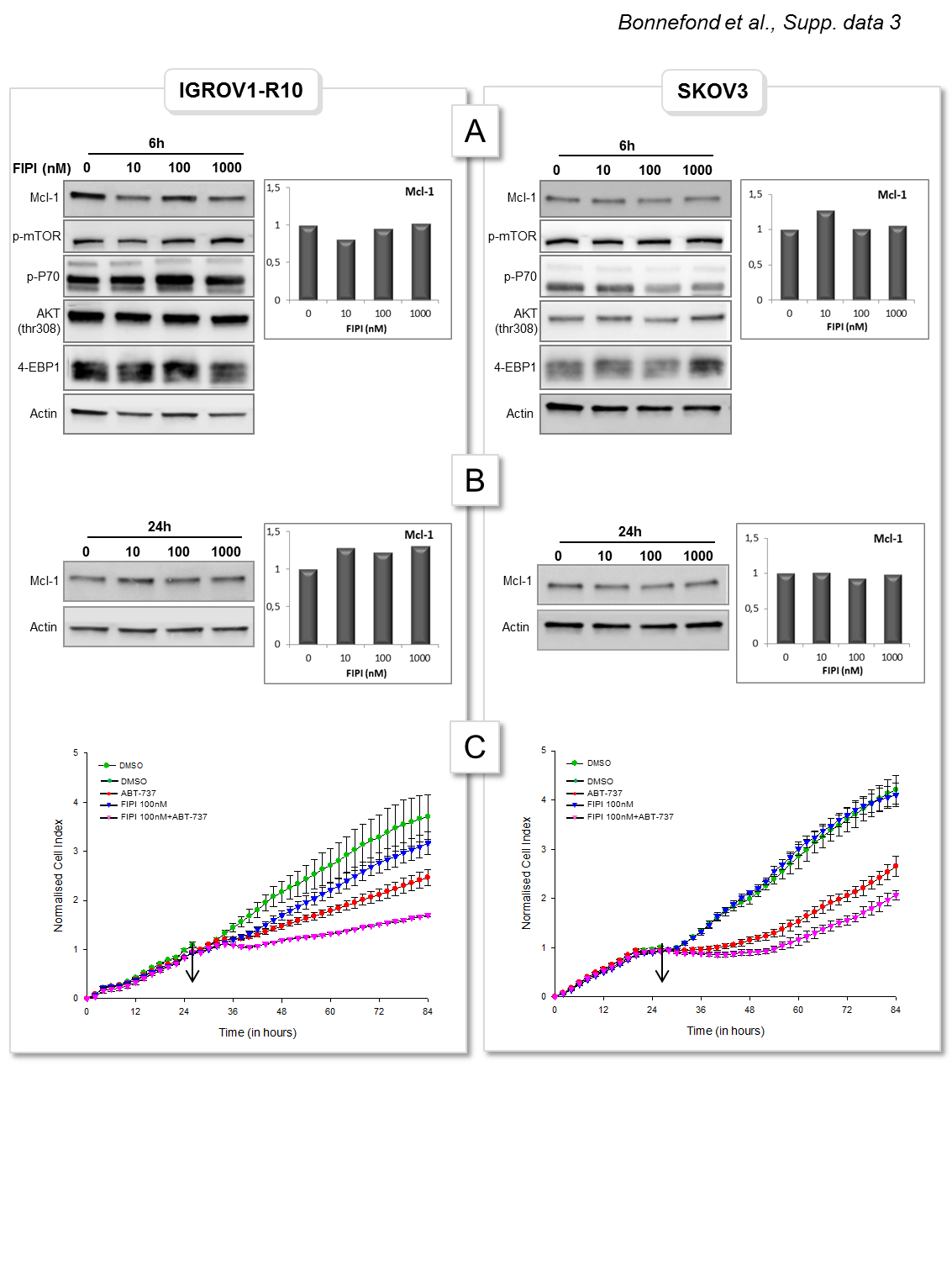

Supplement: Supplementary file 3 — Supplementary material 3 (TIFF 329 kb) [file 10495_2015_1095_MOESM3_ESM.tif]

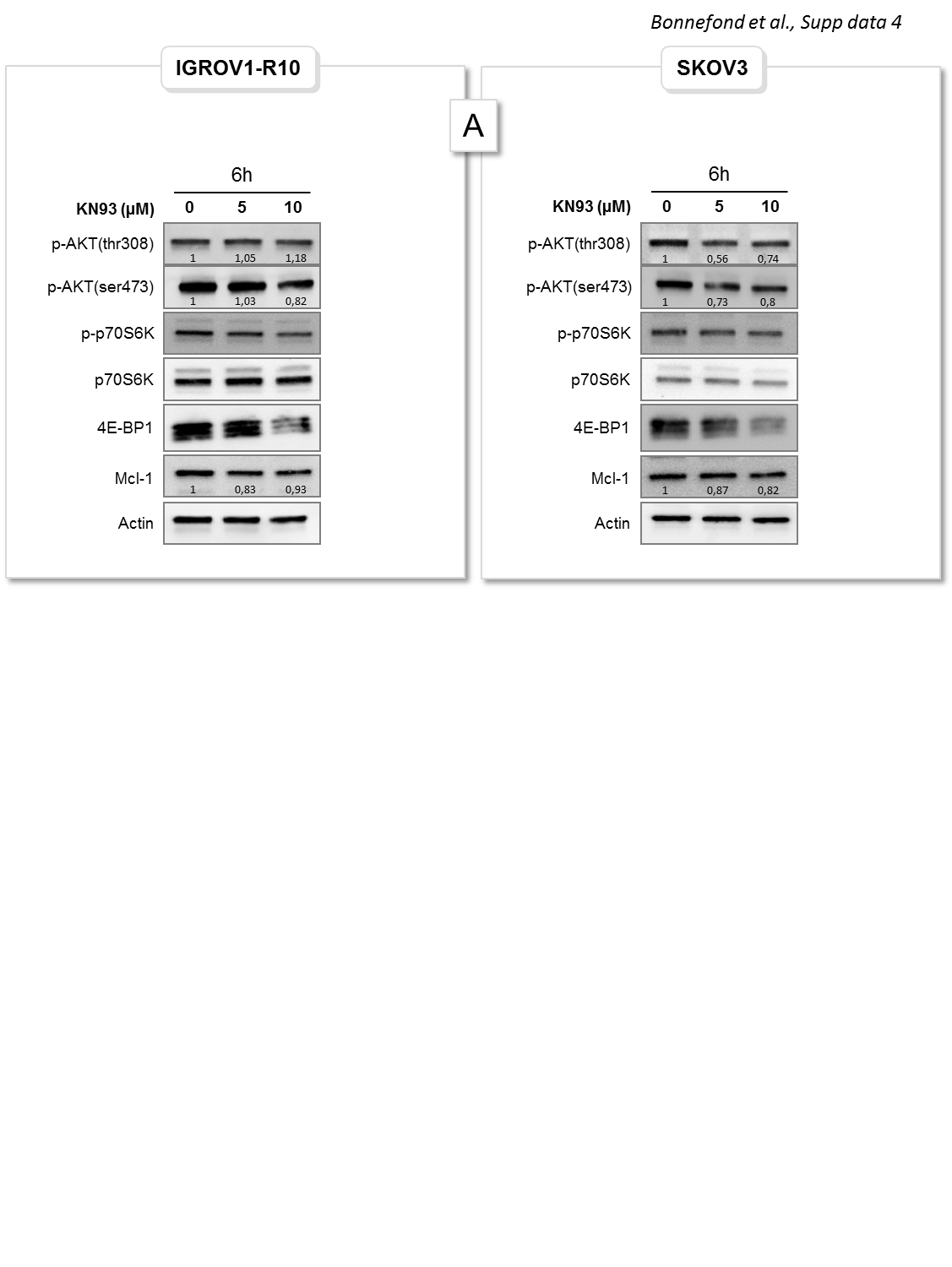

Supplement: Supplementary file 4 — Supplementary material 4 (TIFF 198 kb) [file 10495_2015_1095_MOESM4_ESM.tif]
