# Supplementary material for: Calcium signals inhibition sensitizes ovarian carcinoma cells to anti-Bcl-xL strategies through Mcl-1 down-regulation
Source: Apoptosis. 2015 Jan 28;20(4):535–50. doi: 10.1007/s10495-015-1095-3 (PMC4348506; doi:10.1007/s10495-015-1095-3)
Supplement: Supplementary file 5 — Supplementary material 5 (DOCX 13 kb) [file 10495_2015_1095_MOESM5_ESM.docx]

Supplementary data 1: Calcium chelation combined with siRNA targeting Bcl-x_L_ leads to apoptosis in ovarian carcinoma. **(A)** Real time analysis of cellular cytotoxicity of siXL/BAPTA-AM combination. Histogram was obtained using the xCELLigence System as described in *Material and Methods*. Cells were grown for 24h and then transfected with an ineffective siRNA (siCT) or siXL (arrow). Twenty-four hours after transfection, cells were treated or not (DMSO) with 10 µM BAPTA-AM (dotted arrow). Cell Index was recorded every 2 hours. The results are the means of three replicates, with displayed standard error bars. IGROV1-R10 and SKOV3 cells were transfected or not (Unt) with either siCT or with siXL for 48 hours. After transfection, cells were exposed to 10µM BAPTA-AM or DMSO for 6h. **(B)** Morphological features and **(C)** DNA content were studied for each condition. **(D)** Cell viability was assessed by trypan blue exclusion. **(E)** PARP and caspase 3 cleavages, Mcl-1 and Bcl-x_L_ expressions were studied by western-blot. Data are representative of three independent experiments.

Supplementary data 2: Caspases inhibition prevents BAPTA-AM/ABT-737 combination-induced apoptosis. SKOV3 cells were pre-treated 1h30 with the pan caspase inhibitor z-VAD (50µM) then cotreated with either DMSO or 10µM ABT-737 for 6 hours. **(A)** DNA contents were analysed for each conditions by flow cytometry. **(B)** Cleavages of PARP and Caspase 3 were assessed by western blot.

Supplementary data 3: PLD inhibition does not down-regulate Mcl-1 expression. IGROV1-R10 and SKOV3 were treated for 6h with FIPI, a pharmacological PLD inhibitor. **(A)** Mcl-1, p-mTOR, 4E-BP1, p-P70s6K, and p-AKT(thr308) expressions were assessed by western blot and Mcl-1 expression was quantified by densitometry. **(B)** IGROV1-R10 and SKOV3 were treated for 24h and Mcl-1 expression was assessed by western blot and quantified by densitometry. **(C)** Real time analysis of cellular cytotoxicity of FIPI/ABT-737 combination. Histogram was obtained using the xCELLigence System as described in *Material and Methods*. Cells were grown for 24h and then treated with 100nM FIPI and 10µM ABT-737 (arrow). Cell Index was recorded every 2 hours. The results are the means of three replicates, with displayed standard error bars.

Supplementary data 4: CamKII inhibition does not down-regulate Mcl-1 expression in ovarian carcinoma cells. IGROV1-R10 and SKOV3 were treated for 6h with increasing concentration of KN93, a pharmacological CamKII inhibitor. AKT, p70s6K, 4E-BP1 phosphorylations and Mcl–1 expression were assessed by western blot and proteins expressions were quantified by Image J software. Data are representative of three independent experiments.
